# Macular Thickness Assessment in Patients with Glaucoma and Its Correlation with Visual Fields

**DOI:** 10.5005/jp-journals-10008-1207

**Published:** 2016-10-29

**Authors:** Mafalda Mota, Fernando T Vaz, Mário Ramalho, Catarina Pedrosa, Maria Lisboa, Paulo Kaku, Florindo Esperancinha

**Affiliations:** 1 Resident, Department of Ophthalmology, Prof. Doutor Fernando Fonseca Hospital, Lisbon, Portugal; 2 Ophthalmologist, Department of Ophthalmology, Prof. Doutor Fernando Fonseca Hospital, Lisbon, Portugal; 3 Ophthalmologist, Department of Ophthalmology, Prof. Doutor Fernando Fonseca Hospital, Lisbon, Portugal; 4 Resident, Department of Ophthalmology, Prof. Doutor Fernando Fonseca Hospital, Lisbon, Portugal; 5 Ophthalmologist, Department of Ophthalmology, Prof. Doutor Fernando Fonseca Hospital, Lisbon, Portugal; 6 Ophthalmologist, Department of Ophthalmology, Prof. Doutor Fernando Fonseca Hospital, Lisbon, Portugal; 7 Ophthalmologist, Department of Ophthalmology, Prof. Doutor Fernando Fonseca Hospital, Lisbon, Portugal

**Keywords:** Glaucoma, Macula, Optical coherence tomography, RNFL, Visual field.

## Abstract

**Aim:**

To determine the relationship between macular thickness (MT) and visual field (VF) parameters, as well as with changes in the retinal nerve fiber layer (RNFL) thickness in patients with glaucoma and ocular hypertension (OH).

**Materials and methods:**

Cross-sectional statistical analysis of spectral domain optical coherence tomography (SD-OCT) compared with several VF parameters (mean defect - MD and loss variance - LV), in a nonrandom sample of 70 eyes from patients with glaucoma or OH. Statistical analysis was performed using Statistical Package for Social Sciences^®^. The correlation coefficient used was determined by Spearman correlation and the value of p < 0.05 was considered statistically significant.

**Results:**

A significant correlation was seen between VF parameters and decrease in MT (MD: r = –0.363, p = 0.002; LV: r=–0.378, p = 0.001). The results were more significant when we compared the LV in the group with average MT 270 to 300 μm (r = –0.413, p = 0.015). Asymmetry between the superior macula and inferior macula correlated with LV (r = 0.432, p = 0.019) in the group with MT < 270 μm. There was also a significant correlation between thinning of superior-temporal and inferior-temporal RNFL and the decrease of the superior and inferior MT respectively (p < 0.001).

**Conclusion:**

Spectral domain optical coherence tomography measurements of retinal thickness in the macula correlate with VF parameters and RNFL parameters in glaucoma patients. This relationship was first demonstrated with static computerized perimetry made with Octopus 101^®^. These results can be a valuable aid for evaluating and monitoring of glaucoma patients, establishing a correlation between structure and function. Measurements of retinal thickness in the macula may be an additional instrument for early detection of structural changes and its correlation with functional defects.

**How to cite this article:**

Mota M, Vaz FT, Ramalho M, Pedrosa C, Lisboa M, Kaku P, Esperancinha F. Macular Thickness Assessment in Patients with Glaucoma and Its Correlation with Visual Fields. J Curr Glaucoma Pract 2016;10(3):85-90.

## INTRODUCTION

Glaucoma is a multifactorial chronic optic neuropathy characterized by a gradual loss of retinal ganglion cells together with its axons, the retinal nerve fiber layer (RNFL), leading to typical morphological changes in the optic nerve and possible modification of the visual field (VF). If not detected and treated in early stages, glaucoma might result in irreversible blindness.^1-3^ It is known that structural damage with loss of retinal ganglion cells and their axons precedes functional damage, manifested by typical VF alterations.^4,5^ Structural loss may occur over 5 years before the onset of functional damage, and it is thought that a 35 to 50% loss of RNFL is necessary for alterations in the VF to be detected.^1^ Considering the time lapse between structural and functional damage, it is important to develop a strategy that would allow earlier detection of glaucoma.

About 30 to 50% of retinal ganglion cells are located in the macular area, where these cells are arranged in parallel below the nerve fiber layer (NFL) and correspond to 30 to 35% of the macular thickness (MT).^1,6^ The NFL consists of axons from ganglion cells, glia cells, and astrocytes. This layer becomes thicker as it approaches the optic disk, being the largest layer of the peripapillary retina.^6^ In glaucoma patients, decrease of MT might be attributed to the death of ganglion cells and its axons, ultimately resulting in a decrease in both layers.

Increased resolution imaging with fine detail of retinal layers is now possible using spectral domain optical coherence tomography (SD-OCT). This technique enables macula and peripapillary RNFL analyses, being a valuable asset in diagnosing and monitoring glaucomatous disease. Spectral domain optical coherence tomography is a noninvasive and a no-contact exam widely used for the detection of retina alterations.

Several studies have tried to develop strategies based on structural defects and their correlation with functional defects, to predict and prevent the deterioration of VF in glaucoma patients.^7^ Therefore, this is an area of current research and with enormous potential and benefits for patients with glaucoma.

In this study, we propose to evaluate whether MT is correlated with VF parameters as well as with RNFL changes, in patients with glaucoma and ocular hypertension (OH).

## MATERIALS AND METHODS

Observational and cross-sectional study of 70 eyes of 54 patients diagnosed with glaucoma or OH (nonrandom sampling) attending the Glaucoma Department in Prof. Dr. Fernando Fonseca Hospital (HFF), Lisbon, Portugal, between April and August 2014. All patients underwent SD-OCT and assessment of VF by static computerized perimetry. The tests were preferably made on the same day or with a maximum time lag of 1 month. Based on the total macular thickness (TMT) obtained by SD-OCT, we subdivided the sample into two arbitrarily groups: (1) TMT < 270 μm and (2) TMT between 270 and 300 μm.

We intended to determine the following:

- Correlation between TMT and VF: Analysis of two parameters in VF - mean defect (MD) and loss variance (LV).
- Correlation between asymmetry in superior and inferior MT and LV.
- Correlation between superior *vs.* inferior MT and the thinning of superiotemporal and inferiotemporal RNFL respectively.

### Inclusion Criteria

This study included patients over 18 years of age diagnosed with glaucoma or OH and able to give an informed and free consent. This study followed the tenets of the Declaration of Helsinki and the protocol was approved by the HFF ethics committee.

### Exclusion Criteria

This study excluded potential participants with coexistence of any other significant ocular pathologies that might bias retina thickness measurements or VF parameters, such as the following: Eye media opacities, diabetic retinopathy, aged macular degeneration, or other retinopathies. We also excluded all patients with poor quality VF and OCT (specified characteristics below).

### Spectral Domain Optical Coherence Tomography

The SD-OCT SPECTRALIS^®^ (Heidelberg Engineering GmbH, Germany) was used to study the macula and retina peripapillary area. Spectral domain optical coherence tomography uses confocal laser scanning for image acquisition, by focusing the laser beam on the retina. Two different light beams are used simultaneously to obtain three-dimensional volume scans. These beams are deflected through oscillating mirrors, which allows a sequential scan of the retina and the creation of high-resolution spatial images with a scanning proximity of 11 μm. It is possible to compare the volume and thickness of the retina layers, by comparing the values obtained from an individual with a normative database of normal individuals. This device also incorporates active tracking system of eye movement (TruTrack) and fovea-disk alignment technology (FoDi): It captures two images in the same position, which compensates for the patients' eye and head movement.

Macula assessments were conducted using the macular assessment protocol *“p. pole,”* which encompasses a 30 × 25° scan with 61 scans per section. The default position was in the center, yielding a 8 × 8 grid. Then TMT was evaluated with its superior and inferior subdivisions. All images obtained had a quality above 15.

The RNFL thickness was analyzed for optical disk evaluation, using the glaucoma protocol for “RNFL” evaluation with a scanning pattern of 12° centered on the head of the optical disk. The default pattern position was 2.6° nasal and 2.1° superior from the fovea, and only one scanning per section was performed. The global thickness and six areas of the peripapillary region (temporal – T, inferior-temporal – IT, inferior-nasal – IN, nasal – N, superior-nasal – SN, and superior-temporal – ST) were analyzed using a STINT pattern. Three images were collected with a quality above 15 and “ART 100 frames” of 100%. The best quality image was always selected.

### Static Computerized Perimetry

Visual field analysis was done by SCP technique, using Octopus 101^®^ (Haag-Streit International, Switzerland). The basic parameters used were: Room with white lighting 1.27 or 10 cd/m^2^, a white stimulus with a diameter of 0.43° (corresponding to the III size in Goldmann perimetry), and a time of exposure 100 ms.^4,8,9^ Collected data were used for statistical analysis parameters: MD and LV. Mean defect measures the difference between the normal values, adjusted for the patient's age, and the sensitivity values of the patient. An MD increase would reflect a sensitivity decrease, thus measuring diffuse VF changes in relation to what is considered normal. Loss variance corresponds to the variance of the sensitivity determination thresholds, where increased LV indicates areas with different sensitivity thresholds, i.e., focal or localized defects.^5^ Learning effect is frequent in this approach, two or more exams often required to establish a reliable test base.^4,5^ False positive and false negative of all performed exams were below 33%.

### Statistical Analysis

Statistical analysis was done using the Statistical Package for Social Sciences (SPSS^®^), version 22 (SPSS, Inc., Chicago, IL, USA). Statistical correlations were stabilized using the Spearman correlation. Tests were considered statistically significant for α = 0.05.

## RESULTS

We observed that within the sample population (70 eyes diagnosed with glaucoma or OH, with patients from 18 to 88 years), there was a predominance of male patients (60%), with an average of 67.46 ± 16.57 years (Table 1). The three most common diagnoses were primary open-angle glaucoma (37.14%), pseudo-exfoliative glaucoma (20%), and juvenile glaucoma (11.43%). The least frequent diagnosis was uveitic glaucoma (1.43%).

**Fig. 1:**
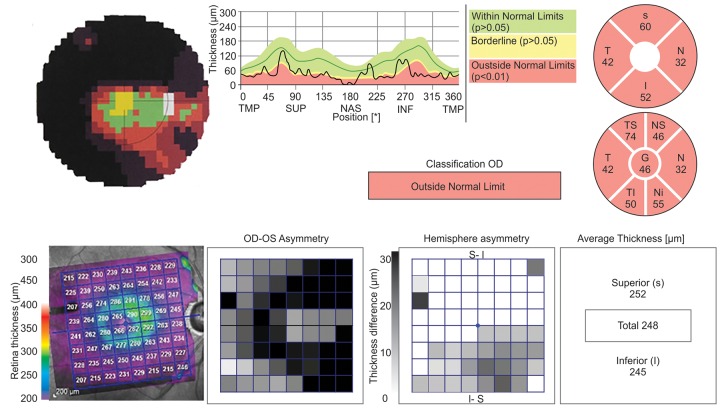
Example obtained through the analysis of SCP and SD-OCT, using the RNFL and p. pole software, in the right eye of a patient with glaucoma. In VF a tubular defect is observed. There is an overall thinning of the RNFL except in inferior-nasal quadrant, which is borderline. In the evaluation by p. pole program (below) we see the asymmetry between superior and inferior macula, which is consistent with the VF obtained from the SCP

Based on the TMT obtained with SD-OCT (Fig. 1), the sample was subdivided into two groups: TMT < 270 μm and TMT between 270 and 300 μm. Some patients displayed TMT > 300 μm (n = 7). We chose not to pursue any statistical analysis regarding the later, since the sample was very reduced and the certainty of any conclusions associated with these analyses would be quite low. The results obtained are summarized in Table 2. The asymmetry between superior and inferior MT was calculated by subtracting the two corresponding values (Table 2).

**Table 1:** **Table 1:** Demographic data

*Gender*			
Female		28 (40%)	
Male		42 (60%)	
*Diagnosis*			
Glaucoma and congenital cataract		2 (2.68%)	
Juvenil glaucoma		8 (11.43%)	
Uveitic glaucoma		1 (1.43%)	
Open angle primary glaucoma		26 (37.14%)	
Close angle primary glaucoma		5 (7.14%)	
Normotensional glaucoma		6 (8.57%)	
Pseudo-exfoliative glaucoma		14 (20%)	
Ocular hypertension		6 (8.57%)	
Pigmentary glaucoma		2 (2.68%)	

**Table 2:** **Table 2:** Median values obtained by OCT and SCP

		*n*		*Median*		*Minimum*		*Maximum*	
Total MT		70		273		219		319	
<270 μm		29		259		219		269	
270-300 μm		34		278.5		270		299	
Superior MT		70		272		213		321	
<270 μm		29		262		213		284	
270-300 μm		34		279		263		300	
Inferior MT		70		272		224		317	
<270 μm		29		259		224		276	
270-300 μm		34		281		265		302	
Asymmetry		70		6		0		33	
<270 μm		29		5		1		33	
270-300 μm		34		7		0		24	
ST RNFL		70		94		32		177	
<270 μm		29		66		32		177	
270-300 μm		34		98		43		163	
IT RNFL		70		94		27		173	
<270 μm		29		61		27		140	
270-300 μm		34		107		34		173	
MD		70		3.3		–3.6		23.7	
<270 μm		29		6.7		–0.9		23.7	
270-300 μm		34		2.05		–3.6		19.2	
LV		70		16.95		1.4		166.1	
<270 μm		29		31.4		1.4		120.9	
270-300 μm		34		10.6		1.6		89.2	

Legend: OCT: Optical coherence tomography; SCP: Static perimetry computed; MT: Macular thickness; ST: Supero-temporal; RNFL: Retinal nerve fiber layer; IT: Infero-temporal; MD: Mean defect; LV: Loss variance

**Graphs 1A and B:**
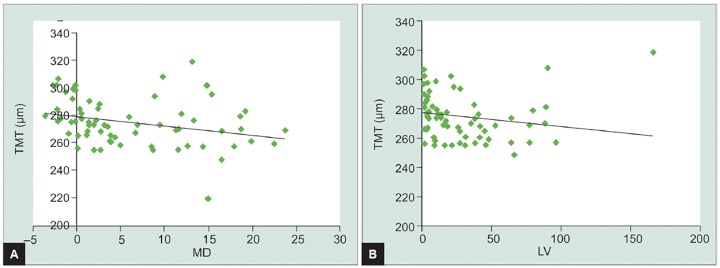
Correlation between TMT and MD (top) and between the TMT and LV (below)

### Correlation between TMT and VF

We found an overall statistically significant negative correlation between the TMT and MD (r=–0.363, p = 0.002), as well as between TMT and LV (r = –0.378, p = 0.001) (Graphs 1A and B).

We further assessed the relationship between TMT, MD and LV within TMT <270 μm and TMT between 270 and 300 μm groups. In TMT < 270 μm group, there was a nonsignificant correlation between TMT and MD as well as between TMT and LV (r=–0.105 MD, p = 0.589; r=–0.177 LV p = 0.357). When evaluating these parameters within patients with TMT between 270 and 300 μm, there was a nonsignificant correlation between TMT and MD (r = –0.265, p = 0.131), but a significant negative correlation between the TMT and LV (r = –0.413, p = 0.015). The relationship between TMT and LV is even more evident in patients with TMT between 270 and 300 μm.

### Correlation between Asymmetry in Superior and Inferior MT and LV

**Graph 2:**
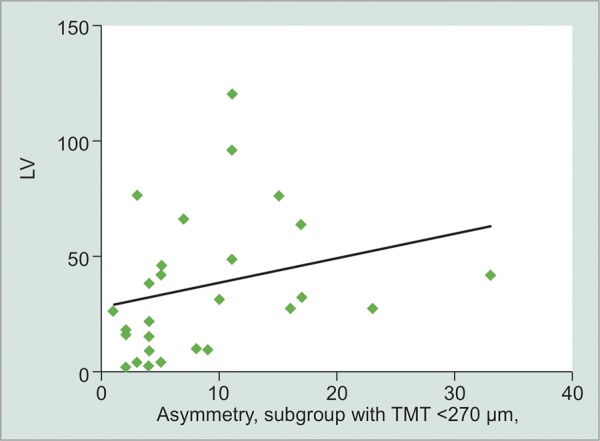
Correlation between LV and MA in the subgroup with EMT < 270 μm

We next explored the relationship between asymmetry in the superior and inferior MT and the LV. We defined macular asymmetry (MA) as the absolute value of the difference between superior and inferior MT. We did not find an overall significant correlation between MA and LV (r = 0.177, p = 0.144). However, there was a significant positive correlation between MA and LV in the TMT < 270 μm group (r = 0.432, p = 0.019, Graph 2). Obtained data indicate that as MA increases, so does LV in patients with a TMT bellow 270 μm.

### Correlation between Superior and Inferior MT Reduction with Thinning of Superiotemporal and Inferiotemporal RNFL

**Graphs 3A and B:**
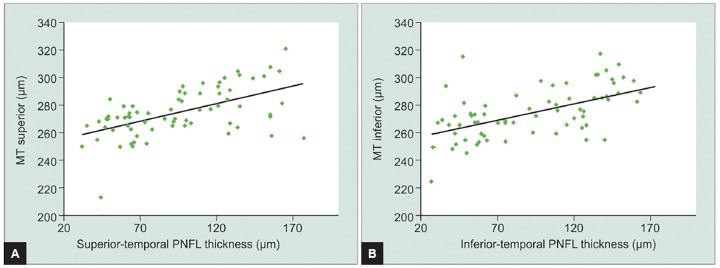
Correlation between superior MT and the thickness and the superiotemporal PNFL, in the top, and between inferior MT and the thickness of inferiotemporal PNFL, below

When comparing the superior MT with thinning of the superiotemporal RNFL, an overall moderate significant positive correlation (r=0.522, p < 0.001, Graphs 3A and B) was observed. We also conducted statistical analysis within both above-mentioned MT groups, but no significant correlations were observed. This indicates that as the superior MT decreases, so does the superiotemporal RNFL thickness.

When analyzing the relationship between inferior MT reduction and inferiotemporal RNFL thinning, again an overall moderate significant positive correlation was detected (r = 0.571, p < 0.001, Graphs 3A and B). Further analysis of TMT < 270 μm and TMT between 270 and 300 μm groups displayed the same trend (r = 0.395, p = 0.034 and r = 0.555, p < 0.001 respectively). This suggests that a decrease in inferior MT seems coupled to a reduction of inferiotemporal RNFL thickness (Graphs 3A and B).

## DISCUSSION

In this study, we proposed to identify parameters of structural RNFL damage associated with glaucoma and OH that would contribute to early diagnosis and subsequent treatment.

First, we uncovered a relationship between MT decrease and VF changes, assessed by a comparison between MT and both MD and LV parameters, using the Octopus 101^®^. Data showed that in general as MT decreases, the MD and VL tend to increase, suggesting that loss of ganglion cells and their axons is functionally translated in diffuse or focal VF defects in patients with glaucoma and OH. This trend was also observed when comparing TMT with LV in the group of TMT between 270 and 300 μm. In contrast, the group with TMT < 270 μm did not display a statistically significant correlation between these parameters. In advanced glaucoma, VF alterations are marked and diffused, meaning that focal changes (i.e., increased LV) might only be detected at earlier stages of the disease. In more advanced stages, characterized by increased cell loss (i.e., lower TMT), focal changes may no longer be detected, potentially explaining the absence of correlation between TMT and both MD and LV.

When observing the asymmetry between the superior and inferior MT and LV, statistically significant results were obtained in the group with TMT < 270 μm, suggesting that in patients with lower TMT, superior and inferior macular asymmetries correlate with focal defects present in the VF. Obtained results point toward macular asymmetries being better observed when the TMT is thinner, reflecting greater damage in the RNFL.

In glaucoma, particularly in earlier stages, there is localized cell loss, mainly in regions, such as the superi-otemporal and inferiotemporal peripapillary quadrants.^10^ We found a moderate significant correlation between the decrease of superior and inferior MT and thinning of the superior and inferiotemporal RNFL. This relationship was also demonstrated when comparing the inferior macula with inferiotemporal RNFL in groups with TMT < 270 μm and TMT between 270 and 300 μm, contrary to the analysis for the superior MT. These results confirm some studies which claim that cellular damage in glaucoma is more severe in inferior macula.^10,11^ Results also suggest that axonal and ganglion cell loss decreases both in the macula and in the peripapillary region and that these changes seem correlated, pointing toward RNFL and macula analysis as important parameters for early diagnosis of glaucoma. The relationship between macular damage and RNFL defects has an increasingly prominent role in glaucoma research, since early detection of retina structural damage is essential for assessing the risk, disease stage, and its progression.^11^ The results presented in this study are consistent with recent studies which evaluated the correlation between structural changes and functional defects in glaucoma.^12,13^

We have encountered some limitations associated with SD-OCT and SCP exams while conducting our analyses. We minimized potential bias factors in the data obtained from SD-OCT, through the acquisition of multiple images for each examination and the choice of images with overall good reliability standards. It is not possible to perform macular stratification layers with our SD-OCT; for this reason we examined the MT. Another possible limitation of this study is that the statistical method used was the Spearman correlation. Additionally, other diseases may cause visible structural changes detectable by SD-OCT, such as macular diseases. We dealt with this potential bias by excluding from this study all patients with other concomitant retinal pathologies. Regarding the SCP, we excluded VFs with low reliability and considered only patients who had already undergone two SCPs, to minimize learning effect bias. Visual field defects (translated by an increased MD) may also be present in other diseases, such as cataract. In order to minimize this potential bias factor, we did not include in this study patients with significant eye media opacification, but included patients with early facoesclerosis.

Spectral domain optical coherence tomography enabled analysis of different retinal layers, particularly of the NFL, the higher layer in the peripapillary region. Efforts are being made for developing algorithms which would process the image obtained by SD-OCT and calculate a range of probabilities of damage assessment, in order to establish predictions on the evolution of glau-comatous disease.^12,13^

## CONCLUSION

Our study demonstrated that, in glaucoma patients, changes in the VF, detected by SCP performed with the Octopus 101^®^, frequently correlate with defects in the macula, evidenced by SD-OCT. Spectral domain optical coherence tomography has the advantage of being a noninvasive test, which is noncontact, low time consuming, and easy to perform. Visual fields are more time-consuming tests and may be difficult to perform. These results could facilitate evaluation and management of patients with glaucoma: Detection of structural changes, diagnosis before functional impairment (early glaucoma diagnosis - preperimetric glaucoma), disease monitoring over time (study progression), and studying the correlation between structural changes and function (perimetric change). The data presented in this study are useful for diagnosis and monitoring of glaucoma in patients in whom VF cannot be performed or when VF results are not reliable.

## References

[1] Greenfield DS, Bagga H, Knighton RW (2003). Macular thickness changes in glaucomatous optic neuropathy detected using optical coherence tomography.. Arch Ophthalmol.

[2] Sommer A, Pollack I, Maumenee AE (1979). Optic disk parameters and onset of glaucomatous field loss. I - Methods and progressive changes in disk morphology.. Arch Ophthalmol.

[3] Pederson JE, Anderson DR (1980). The mode of progressive disk cupping in ocular hypertension and glaucoma.. Arch Ophthalmol.

[4] Quigley HA, Katz J, Derick RJ, Gilbert D, Sommer A (1992). An evaluation of optic disk and NFL examination in monitoring progressive of early glaucoma damage.. Ophthalmology.

[5] Sommer A, Katz J, Quigley HA, Miller NR, Robin AL, Richter RC, Witt KA (1991). Clinically detectable nerve fiber loss and visual field in glaucoma, ischemic neuropathy, papilledema and toxic neuropathy.. Arch Ophthalmol.

[6] Guedes V, Schuman JS, Hertzmark E, Wollstein G, Correnti A, Mancini R, Lederer D, Voskanian S, Velazquez L, Pakter HM (2003). Optical coherence tomography measurement of macular and nerve fiber layer thickness in normal and glaucomatous human eyes.. Ophthalmology.

[7] El Beltagi TA, Bowd C, Boden C, Amini P, Sample PA, Zangwill LM, Weinreb RN (2003). Retinal nerve fiber layer thickness measured with optical coherence tomography is related to visual function in glaucomatous eyes.. Ophthalmology.

[8] Weijland A., Fankhauser F., Bebie H., Flammer J. (2004). Automated perimetry. Visual Field Digest.. HAAG-STREIT AG. V Ed..

[9] Susanna R., Medeiros F. (2005). Perimetria Computorizada, Interpretação e Discussão de Casos. Cultura Médica.. 2° Edição, Rio de Janeiro, Brasil..

[10] Hood DC, Raza AS, de Moraes CG, Liebmann JM, Ritch R (2013). Glaucomatous damage of the macula.. Prog Retin Eye Res.

[11] Park SC, de Moraes CG, Teng CC, Tello C, Liebmann JM, Ritch R (2011). Initial parafoveal versus peripheral scotomas in glaucoma: Risk factors and visual field characteristics.. Ophthalmology.

[12] Mathers K, Rosdahl JA, Asrani S (2014). Correlation of macular thickness with visual fields in glaucoma patients and suspects.. Glaucoma.

[13] Hood DC, Raza AS (2011). Method for comparing visual field defects to local RNFL and RGC damage seen on frequency domain OCT in patients with glaucoma.. Biomed Opt Express.

